# Advancing vaccine manufacturing in Africa: a new era for immunisation programmes towards self-sufficiency

**DOI:** 10.11604/pamj.supp.2025.51.1.46888

**Published:** 2025-06-19

**Authors:** Frankline Sevidzem Wirsiy, Roseline Dzekem Dine, Clovis Nchinjoh Sangwe, Nancy B Tahmo, Eugene Vernyuy Yeika, Clinton Njakoi Kwemu, Jean-Claude Kindzeka Wirsiy, Denis Ebot Ako-Arrey

**Affiliations:** 1 Africa Centres for Disease Control and Prevention (Africa CDC), Addis Ababa, Ethiopia,; 2 Cameroon Baptist Convention Health Board (CBCHB), Yaoundé, Cameroon,; 3 Department of Epidemiology, College of Public Health - University of Nebraska Medical Center (UNMC), Nebraska, USA,; 4 The Pandemic Fund Secretariat, 1818 H Street NW, Washington D.C., USA,; 5 Shoreland Inc. at 933 N. Mayfair Rd., Milwaukee, WI 53226, Wisconsin, USA,; 6 Department of Social Sciences and Community Engagement, Rinda Ubuzima, Kigali, Rwanda,; 7 Department of Health Research Methods, Evidence, and Impact (HEI), McMaster University, Ontario, Canada,; 8 Gavi, the Vaccine Alliance, Geneva, Switzerland,; 9 Division of Public Health, Faculty of Health Sciences, University of the Free State, Bloemfontein, South Africa,; 10 Division of Epidemiology, Dalla Lana School of Public Health, University of Toronto, Toronto, Ontario, Canada,; 11 Health Systems and Programme Manager, Ministry of Public Health, Yaounde, Cameroon,; 12 Biomedical Sciences and Health Program, Faculty of Interdisciplinary at Saint John, University of New Brunswick, New Brunswick, Canada,; 13 Catholic Relief Services (CRS), Brazzaville, Congo

**Keywords:** Vaccine manufacturing, immunisation programmes, Africa

## Abstract

Despite Africa’s impressive vaccination coverage gains over the previous half-century, the continent’s reliance on imported vaccines revealed significant weaknesses in disease control, especially during the COVID-19 pandemic. Establishing a strong and long-lasting vaccine manufacturing infrastructure throughout the continent is essential to achieving health sovereignty and security. The prospects, difficulties, and tactical measures needed to improve vaccine manufacturing in Africa are highlighted in this commentary. Investing in capacity building to create industrial clusters, bolstering regulatory frameworks, and utilizing public-private partnerships are important components. The Partnerships for African Vaccine Manufacturing (PAVM), spearheaded by Africa CDC and the World Health Organization (WHO) support offer blueprints for progress as well as guidelines for advancement. Improving immunisation programmes and getting ready for future public health emergencies depend heavily on achieving vaccine self-sufficiency.

## Commentary

Immunization campaigns in Africa have had a key role in lowering the morbidity and death rates from vaccine-preventable diseases (VPDs), particularly in children. Initiatives like the Expanded Programme on Immunization (EPI) have greatly increased immunization coverage over the last 50 years, especially for illnesses like tetanus, polio, and measles [1]. However, due to a significant reliance on external pharmaceutical companies, the continent still has difficulties guaranteeing fair access to vaccines, which partly contributes to the low vaccination rate in most countries, especially in sub-Saharan Africa (SSA) [2]. As demonstrated during the COVID-19 pandemic, this reliance which supplies more than 99 percent of the vaccinations used in Africa has revealed serious weaknesses in pandemic preparedness and health sovereignty and security [2]. To address this threat, under the Africa CDC, the Partnerships for African Vaccine Manufacturing (PAVM) was founded in 2021 by the Heads of State and Government of the African Union [3]. The primary goal of PAVM is to catalyze the development, production and supply of more than 60% of the vaccine doses needed on the continent by the African Vaccine Manufacturing industry by 2040 [3]. Technically, PAVM is on a multi-stage journey to realize the AU/ Africa CDC´s pillar 3 of the new public health order, which is the “expanded manufacturing of vaccines, diagnostics, and therapeutics to democratize access to life-saving medicines and equipment” [4]. To ensure self-reliance in Africa in the event of a health emergency or outbreak, the partnership has seen the development of multiple promising vaccine manufacturing initiatives, as shown in Table 1. Africa stands at a critical turning point in its chronicles toward vaccine self-sufficiency. The COVID-19 pandemic and other recent outbreaks, such as Mpox, Marburg, and Ebola, have further exposed the continent´s heavy dependence on imported manufactured vaccines, re-iterating the need for a robust local manufacturing ecosystem. This commentary explores key strategies for advancing vaccine production in Africa

**Table 1:** key initiatives supporting vaccine manufacturing in Africa

Initiative	Description	Impact
Africa CDC - Partnerships for African Vaccine Manufacturing (PAVM)	The Africa Centers for Disease Control and Prevention (Africa CDC) is spearheading this program, which offers a strategic road map for expanding the continent's capacity for sustainable vaccine production. By investing, collaborating, and developing infrastructure, PAVM seeks to lessen Africa's reliance on imported vaccinations and promote self-sufficiency.	Increased financial and technical assistance for regional vaccine producers has resulted from the program, which has also aided in the expansion of production facilities throughout several African countries. By 2040, it hopes to reach 60% locally manufactured vaccination coverage and fortifies supply networks.
WHO mRNA Technology Transfer Hub	This hub was set up in South Africa by the World Health Organization (WHO) and other stakeholders to help transfer mRNA vaccine technology to African producers. It makes it possible for regional businesses to create, manufacture, and market mRNA vaccines—including COVID-19 vaccines—without depending on outside patent holders.	By strengthening Africa's capacity to manufacture mRNA-based vaccinations, the hub improves the continent's readiness for pandemics in the future. It supports long-term health security by fostering local proficiency in cutting-edge biotechnology.
African Medicines Agency (AMA)	The AMA is a pan-African regulatory organization whose goal is to standardize medical laws in African countries. It aims to improve pharmaceutical product quality control, expedite clinical trials, and streamline regulatory procedures by establishing a consolidated framework for vaccination approval.	This program ensures that locally produced vaccines satisfy international safety requirements by expediting their clearance. It enhances vaccine distribution efficiency, cuts down on bureaucratic red tape, and fortifies Africa's pharmaceutical regulatory framework.

### The need for local vaccine manufacturing

A lack of local industrial skills has hampered Africa’s capacity to respond to emerging and re-emerging infectious diseases successfully. The manufacture of vaccines is a complex process that requires state-of-the-art equipment, high-level expertise, and strict legal requirements [5]. Increasingly, some African countries, such as Egypt, Senegal, Tunisia, Algeria, Morocco, South Africa, Nigeria, and Ethiopia, have successfully set up centres for vaccine production [2]. Nevertheless, these facilities are not enough to meet the demands of the continent. While the continent strives towards optimizing quality, increasing production capacity is gradually becoming a major issue that needs to be addressed.

### Regional collaboration and Public-Private Partnerships (PPPs)

Public-private partnerships (PPPs) and regional collaboration have effectively tackled health issues worldwide, particularly in Africa. Africa may use these partnerships to boost vaccine manufacturing. Strategic frameworks for encouraging innovation and investment are offered by the Africa CDC’s Partnerships for African Vaccine Manufacturing (PAVM) [3] and the African Vaccine Manufacturing Initiative (AVMI) [6]; which is shedding light on the approach to vaccine self-sufficiency in Africa and assuming the vital function of a reliable information source, particularly through its industry forum. Regional economic blocs like the Southern African Development Community (SADC) and the Economic Community of West African States (ECOWAS) also need to work together to develop shared infrastructure and knowledge.

### Workforce development and capacity building

Building a competent workforce is essential to producing vaccines in a sustainable manner. The availability of local expertise will be guaranteed by agreements with academic institutions and investments in biotechnology training programs, such as the introduction of the International Vaccine Institute with its Africa centre in Rwanda. Additionally, specific capacity-building programs, such as the ones funded by the World Health Organisation (WHO) mRNA technology transfer hub in South Africa, provide templates for increasing production and training [7].

### Overcoming regulatory barriers in Africa

A hurdle to strengthening vaccine manufacturing and access in Africa lies within the continent’s under-resourced and fragmented regulatory landscape. The fact that regulatory systems vary widely in efficiency as well as capacity; across nations warrants that regulatory criteria must be harmonised throughout the African continent to guarantee the quality and safety of locally produced vaccines. The fact that most African national regulatory bodies (NRAs) have not yet achieved WHO Maturity Level 3 (ML3) exacerbates these difficulties. A stable, effective, and integrated regulatory framework is indicated by the achievement of ML3, and the current state of affairs makes it difficult for many nations to guarantee consistent oversight, the safety and quality of medical products, as well as the effective evaluation of novel vaccines and treatments. In particular, by enabling coordinated reviews of clinical trial applications and offering collaborative scientific advice, the African Vaccine Regulatory Forum (AVAREF) had been a trailblazer in this area, greatly accelerating procedures, particularly during public health emergencies like the COVID-19 pandemic. By encouraging cooperation and establishing confidence amongst NRAs, AVAREF laid the crucial foundation for more extensive continental cooperation. Building on these initiatives and striving for a more long-term and all-encompassing answer, this led to the founding of a centralized framework for expediting approval procedures and encouraging cooperation amongst national regulatory bodies; offered by the African Medicines Agency (AMA) [8]. The region will be better equipped to develop vaccines that satisfy global standards thanks to this program. One of the main goals of the Pharmaceutical Manufacturing Plan for Africa (PMPA) [8] is to increase domestic pharmaceutical production, which the AMA will assist with. Additionally, it will be essential in promoting commerce in favor of the Africa Continental Free Trade Area (AfCFTA).

### Policy support and financial investments

Governments, development partners, and the corporate sector must make joint commitments to continue investing in vaccine production. Pooled procurement and advanced market commitments are two examples of creative financing strategies that can lower financial risks and promote long-term investments [9]. Vaccine production must also be given top priority by policymakers in national health and development plans [10]. Lessons from COVID-19 Pandemic, Mpox, Marburg and Ebola outbreaks and what should be the future directions The necessity of vaccination self-reliance has highlighted by the COVID-19 pandemic [10] and further exacerbated by the series of outbreaks such as Mpox, Marburg and Ebola outbreaks. Early in the outbreak of COVID-19, Africa’s inability to obtain enough vaccines made it clear that urgent action was required. In addition to meeting present immunization needs, the creation of continental manufacturing hubs is essential for becoming ready for potential health emergencies requiring vaccination as intervention in the future. It is worthy to note that, from February 4-6, 2025, the Egyptian United Procurement Authority (UPA) held the 2^nd^ Vaccine and Other Health Products Manufacturing Forum for African Union Member States organized by Africa CDC, Gavi, and Regionalized Vaccine Manufacturing Collaborative (RVMC). This was a great chance to hone the strategic vision and direction for regional production of vaccines and other critical health products in Africa.

## Conclusion

In conclusion, increasing vaccine manufacturing in Africa is not only a strategic objective; it is also a pressing need for the sovereignty and health security of the continent. Africa’s excessive reliance on imported vaccinations was brought to light by the COVID-19 pandemic as well as other recent outbreaks such as Mpox, Marburg and Ebola outbreaks, underscoring the urgent need to create a robust and self-sufficient vaccine production ecosystem. While programs like the African Medicines Agency (AMA) and the African Union’s Partnerships for African Vaccine Manufacturing (PAVM) provide encouraging frameworks for advancement, attaining self-reliance will necessitate consistent funding, strong regulatory harmonization, and tactical regional cooperation. Finally, Africa can create a robust vaccine manufacturing industry that can handle both routine immunization demands and future public health emergencies by promoting public-private partnerships, bolstering human capital, and utilizing creative financing methods. With sustained dedication and concerted effort, the target of generating at least 60% of Africa’s vaccine need by 2040 is not merely aspirational; it is attainable. Putting money into domestic vaccine production is an investment in the future economic development, health resiliency, and welfare of future generations of the continent made up of many youths. Africa is poised to usher in a new era of vaccination, one characterized by innovation, self-sufficiency, and fair access to vaccines that can save lives.
